# Fluorescence–phosphorescence dual emissive carbon nitride quantum dots show 25% white emission efficiency enabling single-component WLEDs†
†Electronic supplementary information (ESI) available. See DOI: 10.1039/c9sc03492g


**DOI:** 10.1039/c9sc03492g

**Published:** 2019-08-28

**Authors:** Ting Yuan, Fanglong Yuan, Xiaohong Li, Yunchao Li, Louzhen Fan, Shihe Yang

**Affiliations:** a College of Chemistry , Beijing Normal University , Beijing , 100875 , China . Email: lzfan@bnu.edu.cn; b Guangdong Key Lab of Nano-Micro Material Research , School of Chemical Biology and Biotechnology , Shenzhen Graduate School , Peking University , Shenzhen 518055 , China . Email: chsyang@pku.edu.cn

## Abstract

Developing efficient single-component white light-emitting diodes (WLEDs) is extremely challenging due to the issue of Kasha's rule. Here we report the first demonstration of blue-yellow fluorescence–phosphorescence dual emission from our newly minted single-component white emissive carbon nitride quantum dots (W-CNQDs). The W-CNQDs deliver an overall photoluminescence quantum efficiency of 25%, which is the highest value among white-emitting materials reported to date, based on utilizing both singlet and triplet states. Experimental and theoretical investigations reveal that the carbonyl groups at the rim of the W-CNQDs play a key role in promoting intersystem crossing and inducing intermolecular electronic coupling, affording intensive yellow phosphorescence. Efficient white emission is achieved with a phosphorescence quantum efficiency of 6% under ambient conditions. A WLED is fabricated by integrating W-CNQD phosphors into a UV-LED chip, which shows favorable white light characteristics with CIE coordinates and a CRI of (0.35, 0.39) and 85, respectively, demonstrating good color chromatic stability. This work opens up new opportunities for exploring dual emission mechanisms and designs to facilitate the development of efficient single-component WLEDs.

## Abstract 2

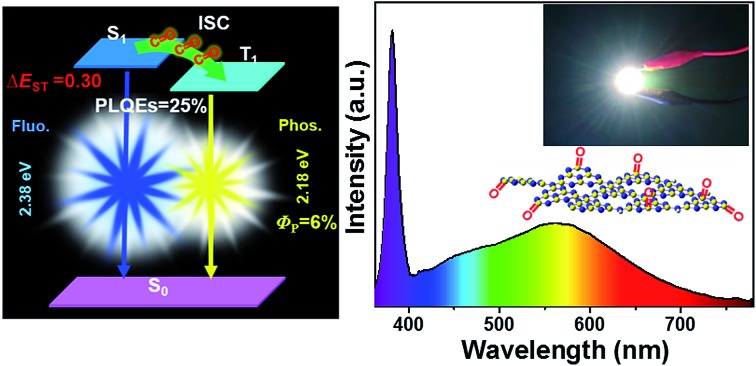
Blue-yellow fluorescence–phosphorescence dual emission from single-component white emissive W-CNQDs with a high PLQE of 25% is reported for the first time.

## Introduction

Single-component material-based white light-emitting diodes (WLEDs), which are aimed at overcoming the color separation and self-absorption problems of mixed emitters, have always been the subject of intense academic research.^1^ An effective strategy for single-component WLEDs is to achieve dual emission by employing singlet and triplet states on account of their sufficient utilization of three quarters of the electrically generated excitons for light emission.^2^ However, it has been a great challenge to develop highly luminescent single-component WLEDs derived from dual emission because of the fundamental limit imposed by Kasha's rule. Specifically, photoluminescence normally originates from only the lowest excited state of a given spin multiplicity, and it is unusual for a single component to make both singlet and triplet states to produce fluorescence–phosphorescence dual emission where complementary colors (*e.g.*, blue and yellow at the very least) are required covering the entire visible spectral window from 400 to 700 nm.^3^ Recently pure organic compounds have been extensively investigated with considerable efforts put into designing and synthesizing dual emissive single-component white light materials by taking advantage of the crystallization-induced phosphorescence mechanism.^4^ However, they often require ordered crystal structures or stringent conditions associated with inert atmospheres to suppress the nonradiative relaxation process of the triplet states, which inevitably results in quenching of white light due to the lack of long-wavelength phosphorescence, arising from undesirable energy transfer between the crystalline and amorphous forms under external environmental changes such as external mechanical stimuli, let alone their high photoluminescence quantum efficiencies (PLQEs) (lower than 10%).^5^ These fatal flaws fundamentally limit their application in disruptive lighting technology. Therefore, highly stable single-component white-light emitters from dual emission with high PLQEs are highly desirable for single-component WLEDs.

The emerging carbon quantum dots (CQDs) have recently been demonstrated to be a superior fluorescence–phosphorescence dual emitter for realizing efficient single-component WLEDs.^6^ However, all of the phosphorescence emissions obtained so far have been limited to blue or green; worse still, they have an extremely low phosphorescence quantum efficiency (*Φ*_P_).^7^ Meanwhile, the synthesis of yellow phosphorescence emissive CQDs for dual emission still remains a major challenge because the necessarily larger size of the sp^2^-conjugation domains and the lack of heteroatoms make them less amenable to spin-flipping between triplet and singlet excited states, but more vulnerable to nonradiative relaxation through thermal and collisional processes.^8^ Thus, without a doubt, developing efficient dual emission with yellow phosphorescence for achieving single-component WLEDs based on white emissive CQDs is highly desirable. Here, we first realize single-component white emission derived from blue-yellow fluorescence–phosphorescence dual emission with an overall PLQE as high as 25% and a relatively high yellow phosphorescence with a *Φ*_P_ of 6% under ambient conditions based on W-CNQDs. Urea, which is widely used for preparing traditional g-C_3_N_4_, was selected as the precursor for synthesizing W-CNQDs.^9^

## Results and discussion

**Fig. 1:**
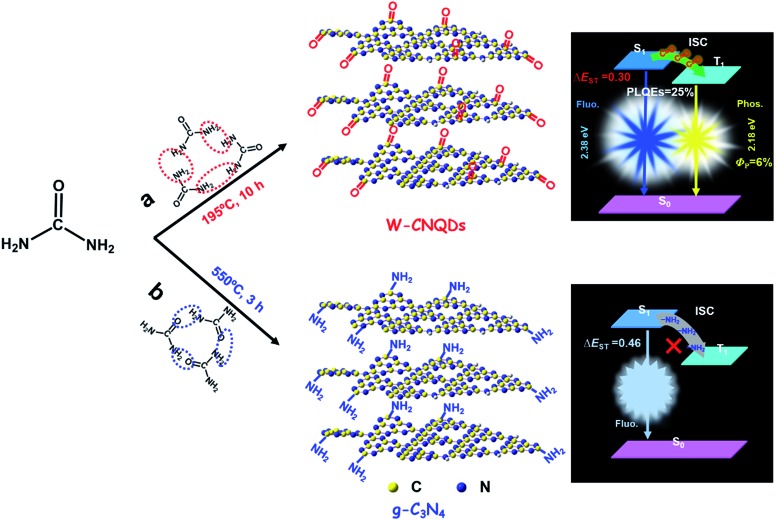
A schematic diagram showing the preparation and energy-level diagrams of the relevant photophysical processes of (a) W-CNQDs and (b) g-C_3_N_4_ (Δ*E*_ST_ = energy gap, S_0_ = ground state, S_1_ = singlet excited state, T_1_ = triplet excited state, ISC = intersystem crossing).

The syntheses of W-CNQDs and traditional g-C_3_N_4_ are comparatively illustrated in Fig. 1. The former involves solid-phase reaction treatment of urea at 195 °C in an autoclave (route a), while the latter takes place at 550 °C in a crucible (route b). The central idea lies in the generation of carbonyl groups (–CO) instead of amino groups (–NH_2_) at the rim of the W-CNQDs under these mild reaction conditions, which plays a key role in promoting intersystem crossing (ISC) and inducing intermolecular electronic coupling, affording intense yellow phosphorescence.^10^ The detailed preparation and purification of the W-CNQDs and traditional g-C_3_N_4_ can be found in ESI (Experimental section, Fig. S1 and S2†).

**Fig. 2:**
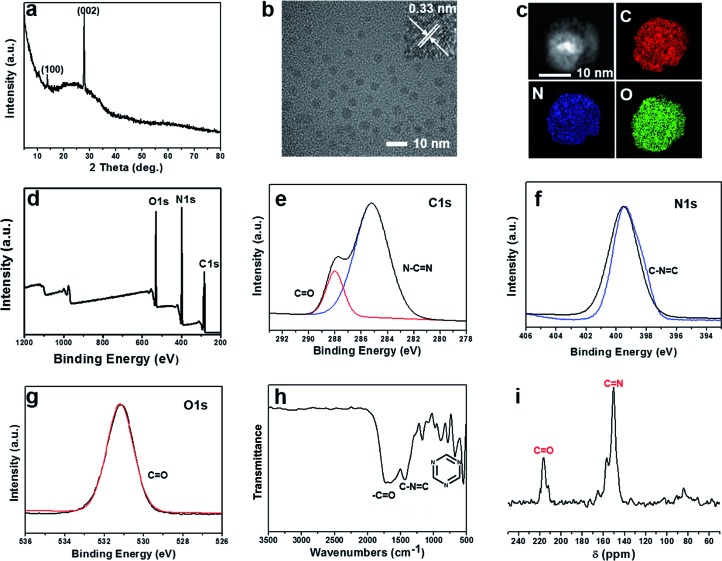
(a) The powder XRD pattern, (b) a TEM image of the W-CNQDs after ultrasonic dispersion in water, (c) elemental mapping images, (d) the XPS survey spectrum and (e) C 1s, (f) N 1s, and (g) O 1s spectra, (h) the FT-IR spectrum, and (i) the solid ^13^C NMR spectrum of the W-CNQDs.

The W-CNQDs were characterized by their X-ray diffraction (XRD) pattern, demonstrating the typical feature of CNQDs with two peaks resulting from the graphite structure and tri-*s*-triazine units in Fig. 2a, which is similar to previous reports. The strongest peak at 27.3° is due to the stacking of the conjugated aromatic system, corresponding to the (002) crystal face, as well as a peak at 13.2°, resulting from the periodic arrangement of the condensed tri-*s*-triazine units, which is indexed as (100).^11^,13b The transmission electron microscopies (TEM) of the W-CNQDs, measured through ultrasonic dispersion in water (Fig. 2b and S3†), reveal that the W-CNQDs are uniform and narrowly distributed around an average size of 5.0 nm. The high-resolution TEM image indicates the high crystallinity of W-CNQDs, and the lattice spacing of 0.33 nm (inset of Fig. 2b) corresponds to the (002) lattice spacing of graphitic carbon nitride.^9^ The elemental ingredients of W-CNQDs were identified by elemental analyses (Table S1†), showing a high oxygen content of ≈18.7% in mass. The elemental mapping images (Fig. 2c) further illustrated the presence and homogeneous distribution of oxygen in W-CNQDs, which are strikingly different from traditional g-C_3_N_4_ with its negligible oxygen content (Fig. S4†), as previously reported.^12^ The high oxygen content of W-CNQDs is further reflected in their X-ray photoelectron spectroscopy (XPS) measurements (Fig. 2d–g). The XPS characterizations (Fig. 2d) reveal that the W-CNQDs mainly contain C, N, O elements with compositions of 38.5%, 43.3%, and 18.3%, respectively, indicating a relatively high amount of doped O in the W-CNQDs compared with traditional g-C_3_N_4_. The high-resolution XPS spectrum of C 1s indicates the presence of C=N (285.2 eV) and C=O (288 eV) bonds in the W-CNQDs (Fig. 2e).^12^ The N 1s spectrum reveals the presence of C–N=C (399.5 eV) (Fig. 2f). The O 1s band contains one peak at 531.2 for C=O (Fig. 2g).^12^ The presence of functional groups in W-CNQDs was also confirmed by the Fourier transform infrared (FT-IR) spectra (Fig. 2h).^12^ The W-CNQDs display FT-IR absorption bands at 790, 1453, and 1720, which can be assigned to the stretching of the triazine ring, C–N=C, and C=O, respectively. Furthermore, solid-state ^13^C-NMR spectra of the W-CNQDs further confirm that the carbonyl groups from the precursor were successfully produced at the rim of the W-CNQD networks. The clearly observable peak located at 210 ppm is indicative of the C=O bond, which fundamentally differs from the negligible resonance signals in the range of 160–220 ppm of the previously reported g-C_3_N_4_ (Fig. S5†). And the peak located at 150–160 ppm may be attributed to the C–N=C bonds, while no signals from saturated sp^3^ carbon atoms were observed (Fig. 2i).^13^ The above detailed characterizations and analyses demonstrate that the carbonyl groups instead of the amino groups were successfully installed at the rim of the W-CNQDs, which is markedly dissimilar to that of the reported traditional g-C_3_N_4_ with the rim being passivated with abundant amino groups. This difference arises from the lower reaction temperature of 195 °C used in the former case, encouraging the loss of NH_3_, as shown in Fig. 1a; whereas in the latter case, the higher temperature of over 550 °C favors the loss of H_2_O, yielding the traditional g-C_3_N_4_, as shown in Fig. 1b.

**Fig. 3:**
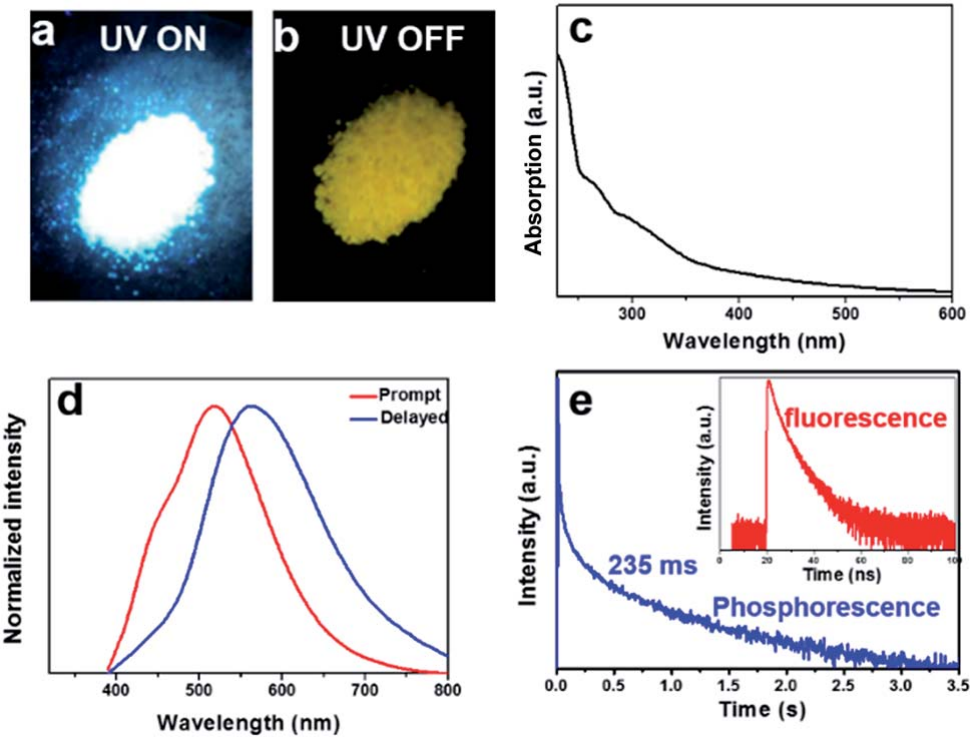
Photographs taken before (a) and after (b) the UV light (365 nm) is turned off. (c) The UV-vis absorption spectrum. (d) Prompt (red line) and delayed (blue line, 5 ms) PL spectra, excited under 380 nm. (e) Time-resolved decay spectra measured at 520 nm for long lifetime and at 440 nm for short lifetime (inset) of W-CNQDs.

The as-prepared W-CNQDs exhibit bright white emission under UV (365 nm) lamp irradiation (Fig. 3a) and yellow phosphorescence emission (Fig. 3b) under ambient conditions when the UV light is turned off. UV/Vis absorption spectra of W-CNQDs powder exhibit two major absorption peaks at around 265 and 320 nm, which are attributed to the π–π* and n–π* transitions of sp^2^ π-conjugation domains and C=O/C=N bonds contained in the W-CNQDs, respectively (Fig. 3c). Fig. 3d shows the prompt PL spectra (red line) of W-CNQDs, which exhibit dual emission bands peaking at around 440 and 520 nm because of the distinctive feature of blue-yellow fluorescence–phosphorescence dual emission. The PL of W-CNQDs can be excited with a wide range of wavelengths and the maximum emission peak position is virtually independent of the excitation wavelength (Fig. S6†). The delayed spectra of W-CNQDs show a single band at 570 nm, revealing that the emission band at 440 nm is a short-lived emission. Therefore, the short-lived and long-lived species are both involved in their light emission process. To confirm this, the time-resolved decay curves at 440 and 520 nm were recorded (Fig. 3e), which reveal the presence of both short- and long-lived species with lifetimes of 3.8 ns and 235 ms under ambient conditions, respectively. The fluorescence emission is fitted with a double-exponential decay; the phosphorescent part can be fitted with a three-exponential decay. The overall absolute PLQE of W-CNQDs is 25%, and the *Φ*_P_ of yellow phosphorescence reaches 6% under ambient conditions. From these data, the dynamic photophysical parameters of both fluorescence and phosphorescence from W-CNQDs were extrapolated (Table S2†). The ISC rates (*k*_isc_ = 1.6 × 10^7^) of W-CNQDs are comparable to their corresponding radiative decay rates (*k*^F^ = 6.6 × 10^7^), indicating an efficient ISC process to produce sufficient triplet excitons responsible for the efficient phosphorescence with a *Φ*_P_ of 6% under ambient conditions, which is indeed the highest reported so far, thus enabling efficient white emission. In addition, typically small singlet–triplet energy gaps (Δ*E*_ST_ < 0.4 eV) are required to facilitate the ISC process in phosphorescence emitters;^15^ therefore, the low-temperature fluorescence and phosphorescence spectra at 77 K of the W-CNQDs were measured to estimate Δ*E*_ST_ as 0.30 eV, which is far less than that of traditional g-C_3_N_4_ (Δ*E*_ST_ = 0.46 eV) (Fig. S7 and S8†). Such a small Δ*E*_ST_ should be closely related to the carbonyl groups at the rim of the W-CNQDs, facilitating the ISC process and hence efficient triplet-exciton generation.

**Fig. 4:**
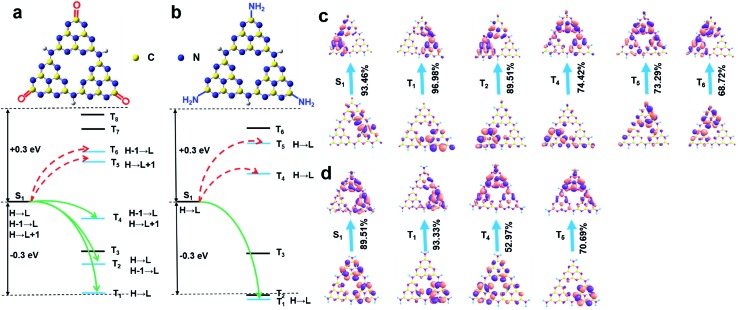
Schematic representations of the TD-DFT calculated energy levels, main orbital configurations, and possible ISC channels of (a) W-CNQDs, and (b) g-C_3_N_4_ in the singlet (S_1_) and triplet (T_*n*_) states. H and L refer to the HOMO and LUMO, respectively. Diagrams showing NTOs (hole orbitals on the bottom and electron orbitals on the top) and the corresponding proportions for (c) W-CNQDs and (d) g-C_3_N_4_.

Taken together, it is evident that at the low reaction temperature, the carbonyl groups instead of amino groups were anchored at the rim of the W-CNQDs, which is key to enhancing ISC for yellow phosphorescence emission. TD-DFT theory calculations on the W-CNQDs and traditional g-C_3_N_4_ for comparison were performed to confirm the mechanism above (Fig. S9 and S10†). The calculated Δ*E*_ST_ of the W-CNQDs is 0.32 eV (*E*_S_1__ = 3.4271 eV, *E*_T_1__ = 3.1071 eV), which is smaller than those of traditional g-C_3_N_4_ (*E*_S_1__ = 3.5043 eV, *E*_T_1__ = 3.0843 eV, Δ*E*_ST_ = 0.42 eV), which potentially enables effective ISC from S_1_ to T_1_ states (Tables S3 and S4†).^14^ And this small Δ*E*_ST_ value (Δ*E*_ST_ = 0.32 eV) of the W-CNQDs based on theoretical calculation is close to the experimental value (Δ*E*_ST_ = 0.30 eV). Furthermore, for the W-CNQDs, the numbers of energy transition channels (five channels) (S_1_ → T_1_, T_2_, T_4_, T_5_, T_6_) increased in comparison to traditional g-C_3_N_4_ (three channels) (S_1_ → T_1_, T_4_, T_5_), also resulting in enhanced ISC (Fig. 4a and b, Tables S3 and S4†).^15^ More significantly, the decreased energy level of the singlet state and the increased energy level of the triplet excited state of the W-CNQDs (*E*_S_1__ = 3.4271 eV and *E*_T_1__ = 3.1071 eV) compared with those of traditional g-C_3_N_4_ (*E*_S_1__ = 3.5043 eV, *E*_T_1__ = 3.0843 eV), also enhance the yellow phosphorescence according to the energy gap law. In addition, we analyzed the characters of the excited states using the natural transition orbitals (NTOs)^16^ for W-CNQDs (Fig. 4c) and traditional g-C_3_N_4_ (Fig. 4d). It is well known that the spatial separation of hole NTOs and electron NTOs can lead to charge transfer characteristics, thus resulting in a small Δ*E*_ST_. The degree of spatial separation of the hole NTOs and electron NTOs can be qualitatively judged by their corresponding electron cloud density distributions around the whole molecular structure. Clearly, as suggested in Fig. 4c, from T_1_, T_2_, T_4_, T_5_, to T_6_, the hole NTO and electron NTO distributions of W-CNQDs show more distinct spatial separation than those of traditional g-C_3_N_4_ (T_1_, T_4_, T_5_) (Fig. 4d), suggesting that the W-CNQDs have charge transfer characteristics, which leads to a small Δ*E*_ST_ and enhanced S_1_ → T_*n*_ ISC. Taking this a step further, we deduce that the carbonyl groups may induce intermolecular electronic coupling within the π-conjugated structure, which enhances the phosphorescence emission as well. Along these lines, the coupled W-CNQDs were investigated to explore the effect of intermolecular electronic coupling on phosphorescence (Fig. S11†). Indeed, the coupled W-CNQDs show a smaller Δ*E*_ST_ (*E*_S_1__ = 3.3946 eV, *E*_T_1__ = 3.1054 eV, Δ*E*_ST_ = 0.29 eV) and increased numbers of energy transition channels (seven channels) (S_1_ → T_1_, T_3_, T_5_, T_6_, T_7_, T_8_, T_9_) in contrast to the isolated W-CNQDs above (five channels), resulting in enhanced ISC (Fig. S12, Table S5†). The role of the carbonyl groups in promoting ISC and inducing the intermolecular electronic coupling believed to be responsible for the intensive phosphorescence emission was further confirmed by control experiments (Fig. S13–S22, Tables S6 and S7†).

**Fig. 5:**
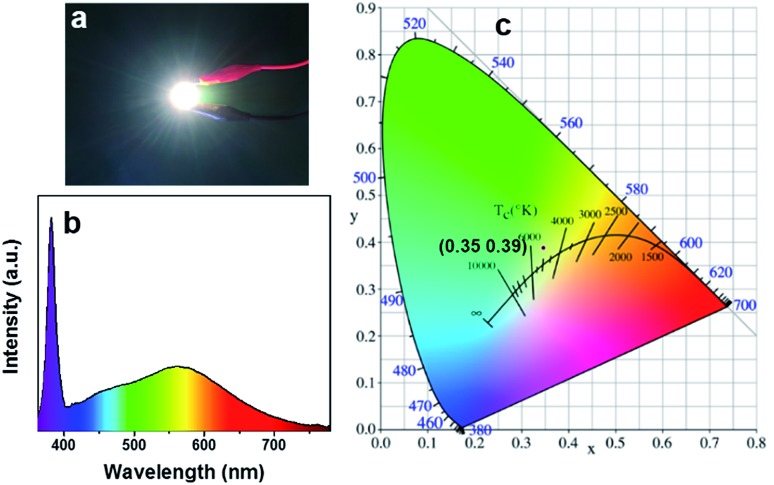
(a) An image of a UV-pumped WLED using the W-CNQDs as phosphors; and the (b) EL spectrum and (c) CIE color coordinates of the WLED lamp.

The highly efficient blue-yellow fluorescence–phosphorescence dual emission of W-CNQDs, coupled with their low cost and environmental-friendliness are compelling for their application in high performance single-component WLEDs. Due to the poor solubility of W-CNQDs, a UV-pumped WLED was fabricated for the first time by directly coating W-CNQD phosphor with cyanoacrylate (Super Glue) onto the surface of a commercial 380 nm UV-LED (Fig. 5a). Efficient white emission from the LED could be observed under a 20 mA forward bias current. Furthermore, the LED showed yellow phosphorescence after the electrical excitation was removed (Fig. S24†). The electroluminescence (EL) spectrum of the as-fabricated efficient single-component WLED based on W-CNQDs phosphors covers a broad spectral region from 380 to 780 nm, as seen in Fig. 5b. Two distinct peaks are clearly observable: one at 450 nm corresponding to blue fluorescence and another at 570 nm originating from the yellow phosphorescence emission. EL spectra show observable red shift compared with that of steady-state PL spectra due to inevitable consecutive reabsorption.^17^ The CIE chromaticity coordinates of the WLED lamp are determined to be (0.35, 0.39) (Fig. 5c), which give a CCT of 4935 K. The CRI value for the WLED based on W-CNQDs is 85, which is even comparable to that of some state-of-the-art single-component WLEDs based on semiconductor QDs and lead halide perovskites (Table S8†). Furthermore, the single-component WLED lamp shows good chromatic stability with an applied current increasing from 20 to 90 mA (Fig. S25–S28†) and high device stability after working for 72 h (Fig. S29–S32†).

## Conclusions

In summary, we report the first successful demonstration of blue-yellow fluorescence–phosphorescence dual emission for single component white light emission with an overall PLQE as high as 25% and a relatively high yellow phosphorescence quantum efficiency of 6% under ambient conditions based on W-CNQDs. The key role of the carbonyl groups at the rim of the large π-conjugated structure in W-CNQDs has been uncovered, assisting the ISC arising from carbonyl (nπ*) mediated intermolecular (ππ*) electronic coupling. A WLED was fabricated by integrating W-CNQD phosphors in a UV-LED chip, which showed favorable white light characteristics with CIE coordinates and a CRI of (0.35, 0.39) and 85, respectively. This work opens up new opportunities for designing single-component WLEDs *via* dual emission strategies. We anticipate that further improving the *Φ*_P_ value of yellow phosphorescence emissive CNQDs and red-shifting their emission will lead to greatly improved performance for carbon-based phosphor single-component WLEDs; this research is underway in our laboratory and will be reported in due course.

## Conflicts of interest

There are no conflicts to declare.

## Supplementary Material

Supplementary information
