# Supplementary material for: Fluorescence–phosphorescence dual emissive carbon nitride quantum dots show 25% white emission efficiency enabling single-component WLEDs
Source: Chem Sci. 2019 Aug 28;10(42):9801–6. doi: 10.1039/c9sc03492g (PMC6993616; doi:10.1039/c9sc03492g)
Supplement: Supplementary file 1 [file SC-010-C9SC03492G-s001.pdf]

Electronic Supplementary Material (ESI) for Chemical Science.
This journal is © The Royal Society of Chemistry 2019

Supporting Information

Fluorescence-Phosphorescence Dual Emissive Carbon Nitride Quantum Dots Scores 25% White Emission Efficiency Enabling Single-Component WLEDs

Ting Yuan [a], Fanglong Yuan [a], Xiaohong Li [a], Yunchao Li [a], Louzhen Fan, [a] Shihe Yang* [b]*

[a] *College of Chemistry, Beijing Normal University, Beijing, 100875, China*

[b] *Guangdong Key Lab of Nano-Micro Material Research, School of Chemical Biology and Biotechnology, Shenzhen Graduate School, Peking University, Shenzhen 518055, China*

I. Synthesis and characterization.

Experimental Section

Synthesis of W-CNQDs: urea(10 mg) was transferred to a poly(tetrafluoroethylene) (Teflon)-lined autoclave (25 mL) and heated at 195 ºC for 10 h. After the reaction, the reactor was cooled to room temperature naturally. The obtained crude W-CNQDs powder were dispersed in deionized water and ethyl alcohol and sonication for 10 min, and was then centrifuged (12,000 rpm/min for 5 min). After several times of the above process, the precipitate was collected and subjected to dialysis (MWCO: 3500 Da) for 6 days. Finally, the W-CNQDs was obtained as a pale yellow powder (ca. 12.6 mg, ~12.6% yield) by freeze-drying.

Synthesis of traditional g-C_3N_4: urea (10 mg) was transferred to a crucible with a cover and heated at 550 ºC for 3 hours to complete the reaction under ambient pressure in air. The obtained crude g-C_3N_4 powder was dispersed in deionized water and ethyl alcohol and sonication for 10 min, and was then centrifuged (12,000 rpm/min for 5 min). After several times of the above process, the precipitate was collected and subjected to dialysis (MWCO: 3500 Da) for 6 days. Finally, the g-C_3N_4 was obtained as a yellow powder (ca. 14 mg, ~14% yield) by freeze-drying.

Synthesis of B-CNQDs: urea(10 mg) was transferred to a poly(tetrafluoroethylene) (Teflon)-lined autoclave (25 mL) and heated at 160 ºC for 10 h. After the reaction, the reactor was cooled to room temperature naturally. The obtained crude B-CNQDs powder were dispersed in deionized water and ethyl alcohol and sonication for 10 min, and was then centrifuged (12,000 rpm/min for 5 min). After several times of the above process, the precipitate was collected and subjected to dialysis (MWCO: 3500 Da) for 6 days. Finally, the W-CNQDs was obtained as a pale yellow powder (ca. 12.8 mg, ~12.8% yield) by freeze-drying.

Characterization Method: A JEOL JEM 2100 transmission electron microscope (TEM) was used to investigate the morphologies of W-CNQDs. The samples of the W-CNQDs and B-CNQDs with or without ultrasonic dispersion in water to measure TEM and elemental mapping images separately. X-ray diffraction (XRD) pattern was carried out by an X-ray diffraction using Cu-Ka radiation (XRD, PANalytical X’Pert Pro

MPD). Absorption spectra were recorded on UV-2600 spectrophotometry. The photoluminescence (PL) spectra and time-resolved emission decay data were obtained using a spectrometer (FLSP980) from Edinburgh Instruments. The absolute overall photoluminescence quantum efficiencies (PLQEs) and phosphorescence quantum efficiencies (Φ_P) were obtained using Edinburgh FLSP980 fluorescence spectrophotometer equipped with a xenon arc lamp (Xe900) and a microsecond flash-lamp (μF900), and an integrating sphere, respectively. The photographs were taken with camera (Nikon, D7200) under UV light excited at 365 nm (UV light: SPECTROLINE, ENF-280C/FBE, 8W). The FT-IR spectrum was measured using a Nicolet 380 spectrograph. X-ray photoelectron spectroscopy (XPS) was performed with an ESCALab220i-XL electron spectrometer from VG Scientific using 300 W Al Ka radiation. ^{13}C solid-state nuclear magnetic resonance (NMR) spectra were determined on a Bruker Advance 300 MHz spectrometer. Elemental analysis was done at VARIO EL (Germany) for determination of the C, N and O contents.

Fabrication of single-component WLEDs: UV-LED chips (LUMEX-SSL-LXTO46UV1C) with the peak emission wavelength centered at 380 nm were used for the fabrication of single-component WLEDs. A certain amount W-CNQDs phosphors was mixed with silicone thoroughly and the obtained phosphor-silicone mixture was coated on the surface of the UV-LED chips to produce single-component WLEDs. The photoelectric properties of the fabricated devices were measured by an integrating sphere spectroradiometer system (LHS-1000, Everfine).

The W-CNQDs show pale yellow appearance (Figure S1) under daylight and bright white emission (Figure 3a) under UV light excitation. The g-C_3N_4 appears yellow under daylight and light blue emission under UV light excitation. (Figure S2).

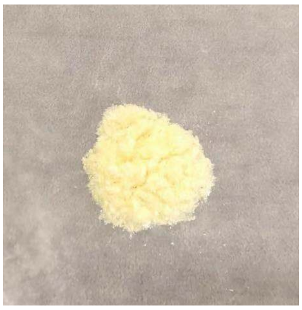

Figure S1. The photographs under daylight of the W-CNQDs powder.

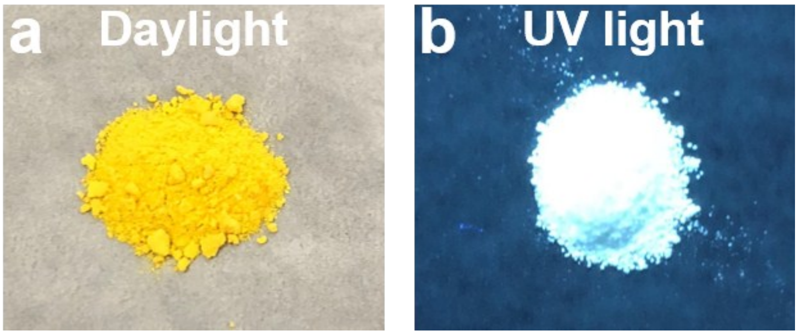

Figure S2. The photographs under daylight (a) and UV light (excited at 365 nm) (b) of the traditional g-C_3N_4 powder.

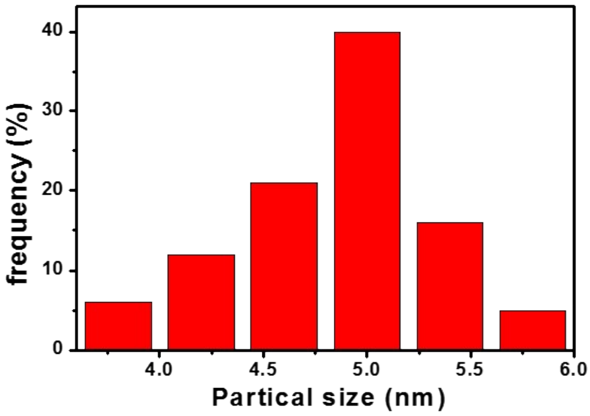

Figure S3. The size distribution of the W-CNQDs.

Table S1 Elemental analysis of the W-CNQDs.

Sample Name	C (wt %)	N (wt %)	H (wt %)	O (wt %)
W-CNQDs	33.8 %	45.1 %	2.4%	18.7%

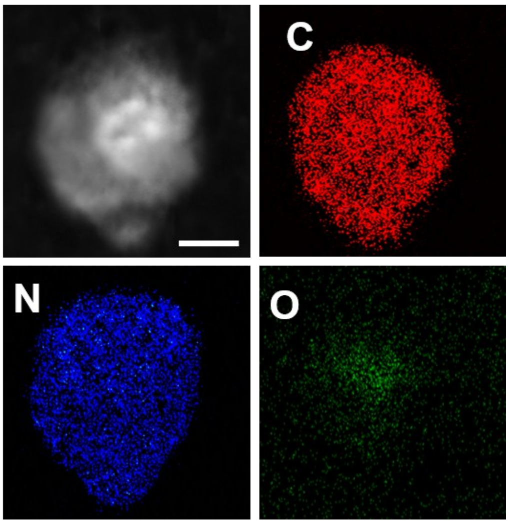

Figure S4 Elemental mapping images of the traditional g-C_3N_4 (scale bar, 20 nm).

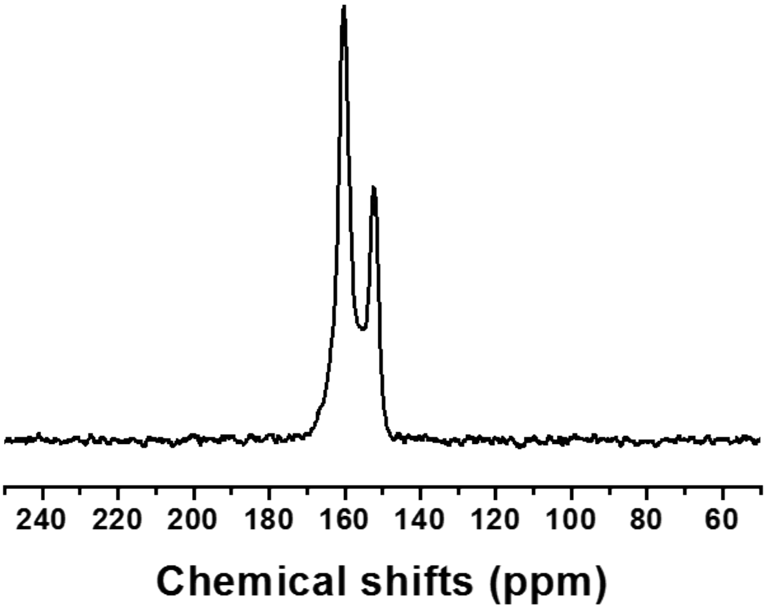

Figure S5 Solid ^{13}CNMR spectrum of the traditional g-C_3N_4.

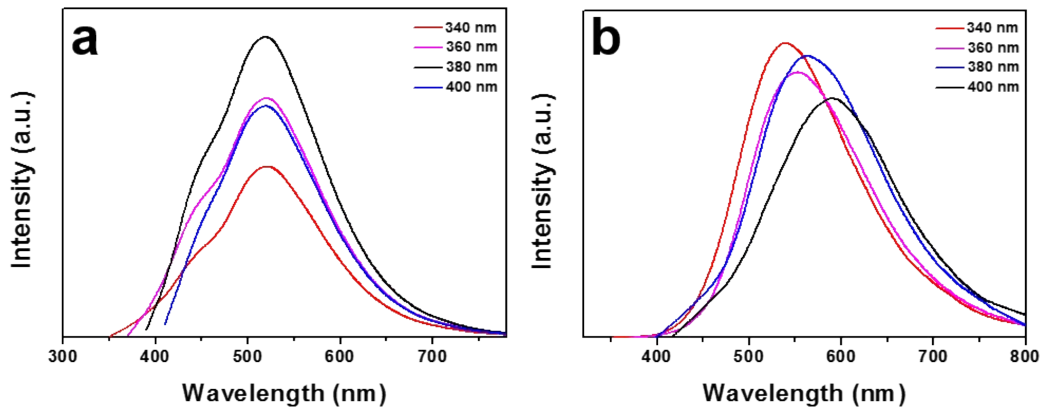

Figure S6. (a) The prompt and (b) delayed (5 ms) PL spectra under different excitation wavelengths of the W-CNQD at 295 K.

Sample	Wavelength (nm)	Fluorescence					Phosphorescence			
		τ_F (ns)	$\emptyset_F$ (%)	k_F (s^{-1})	k_{nr}^F (s^{-1})	k_{ISC} (s^{-1})	τ_P (ms)	$\emptyset_P$ (%)	k_P (s^{-1})	k_{nr}^P (s^{-1})
**W-CNQDs**	**440**<br>**520**	**4.8**	**25**	**6.6** $\times 10^7$	**1.8** $\times 10^8$	**1.6** $\times 10^7$	**235**	**6**	**0.26**	**2.94**

Table S2. Dynamic photophysical parameters of the W-CNQDs.

$k_F = \emptyset_F/\tau_F$; $k_P = \emptyset_P/\tau_P$; $k_{ISC} = \emptyset_P/\tau_F$; $k_{nr}^F = (1 - \emptyset_P - \emptyset_F)/\tau_F$;

$k_{nr}^P = (1 - \emptyset_P - \emptyset_F)/\tau_P$

Typically small singlet-triplet energy gaps (ΔE_{ST}<0.4 eV) are required to facilitate the ISC process in phosphorescence emitters,[1] therefore, the low temperature fluorescence and phosphorescence spectra at 77 K of the W-CNQDs and traditional g-C_3N_4 were measured to estimate ΔE_{ST} (Figure S7-S8).

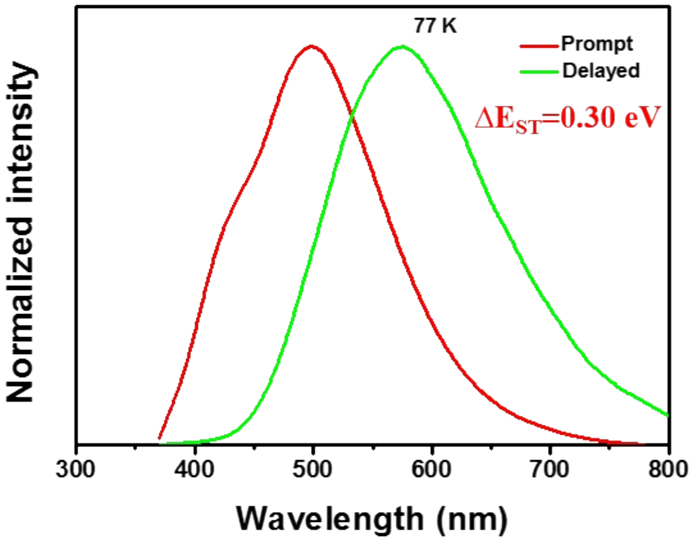

Figure S7. Normalized steady-state photoluminescence spectrum (red line) and delayed photoluminescence spectrum (green line, 5 ms) of the W-CNQDs excited under 370 nm at 77 K.

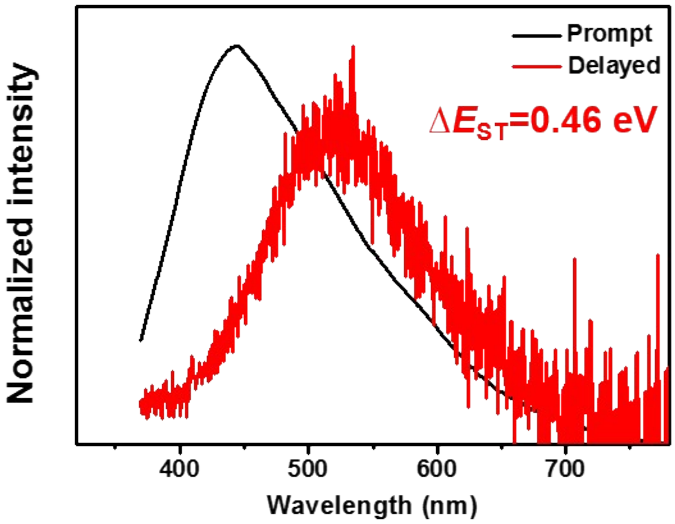

Figure S8. Normalized steady-state photoluminescence spectrum (black line) and delayed photoluminescence spectrum (red line, 5 ms) of the traditional g-C_3N_4 excited under 380 nm at 77 K.

II. Time-dependent density functional theory (TD-DFT) calculations.

TD-DFT calculations were performed on Gaussian 09 program (Revision A. 02). The ground (S_0) and lowest triplet (T_1) state geometries were fully optimized with the Becke's three-parameter exchange functional along with the Lee Yang Parr's correlation functional (B3LYP) using 6-31G (d) basis sets. These optimized stationary points were further characterized by harmonic vibration frequency analysis to ensure that real local minima had been found. The excitation energies in the n-th singlet (S_n) and n-th triplet (T_n) states were obtained using the TD-DFT method based on an optimized molecular structure at ground state (S_0). The possible S_1 to T_n ISC channels are believed to share part of the same transition orbital compositions, and the energy levels of T_n are considered to lie within the range of $E_{S1} \pm 0.3$ or 0.4 eV. Especially, the major intersystem crossing processes (ISC) channels are mainly determined based on two elements. Firstly, the ratio of the same transition configuration in S_1 and T_n should be large in all the transition orbital compositions. Secondly, the energy gap between S_1

and the specific T_n state should be small. When the energy of T_n is lower than S_1, the first element is considered to be more important.[2, 3] The determination of minor ISC channels is vice versa. In the following Table S3 and Table S4, the notations H and L refer to the highest occupied molecular orbital (HOMO) and the lowest unoccupied molecular orbital (LUMO), respectively.

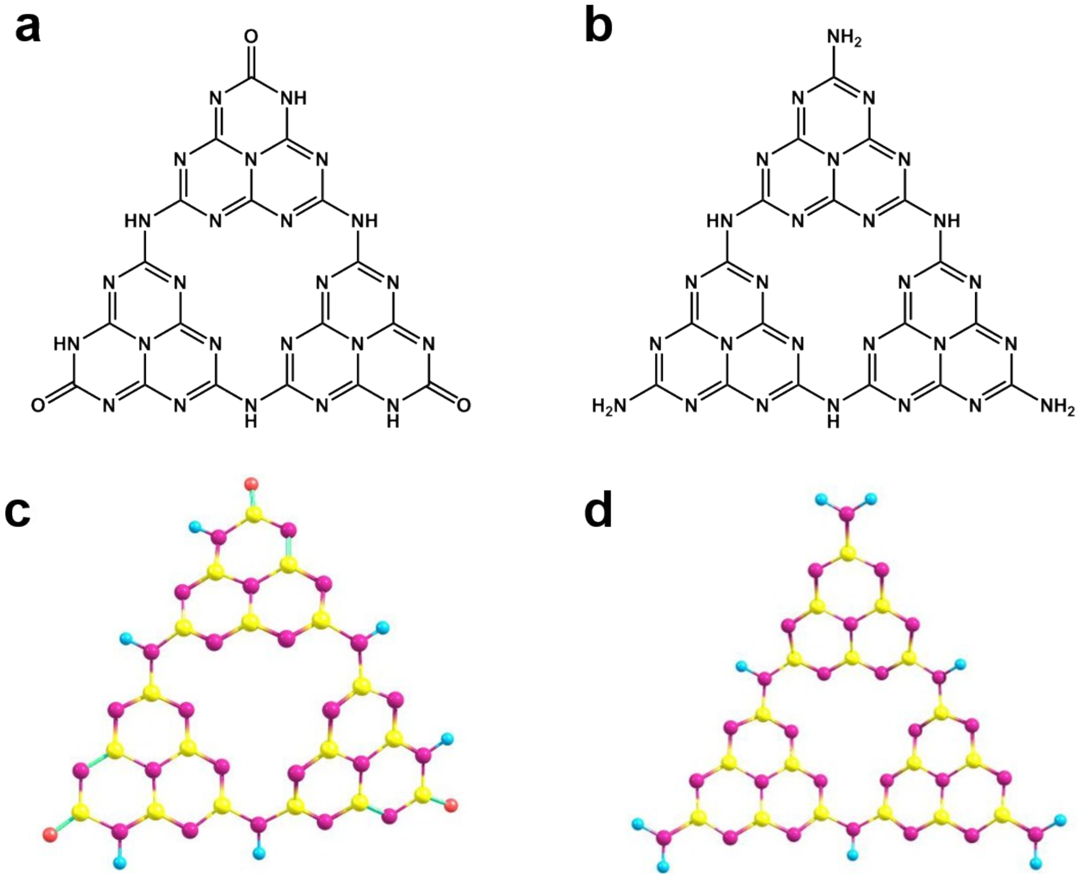

Figure S9. The model of (a) W-CNQDs and (b) traditional g-C_3N_4; optimized geometries of (c) W-CNQDs and (d) g-C_3N_4.

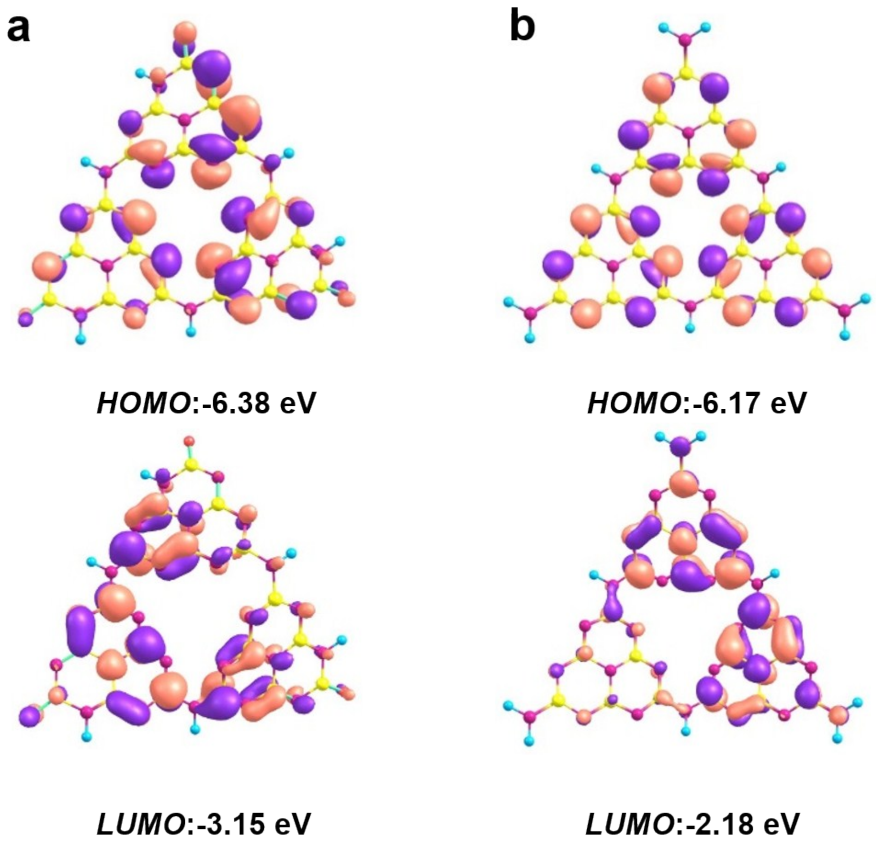

Figure S10. The calculated frontier molecular orbitals for (a) W-CNQDs and (b) traditional g-C_3N_4.

Table S3. The singlet and triplet excited state transition configurations of the W-CNQDs revealed by TD-DFT calculations. The matched excited states that contain the same orbital transition components of S_1 were highlighted in red.

	n-th	Energy (eV)	Transition configuration (%)
S_n	1	3.4271	H-1→L (47.97), H→L (43.51), H→L+1 (11.04)
T_n	1	3.1071	H→L (67.56), H→L+2 (10.39)
	2	3.1835	H-2→L (11.74), H-1→L (14.69), H→L (49.34), H-1→L+1 (42.19)
	3	3.2744	H-4→L (18.50), H-2→L (35.50), H-1→L+1 (25.30), H-1→L+2 (21.12), H→L+2 (13.61)
	4	3.3102	H-4→L+1(15.47), H-2→L (16.83), H-1→L (22.48), H-1→L+1 (55.46), H→L+1 (15.42),
	5	3.6895	H-4→L+1 (10.91), H-3→L (20.82), H-3→L+1 (16.48), H→L+1 (51.50), H-2→L+2 (19.36), H-1→L+2 (15.56), H-1→L+4 (12.14)
	6	3.7011	H-11→L (11.30), H-6→L (23.67), H-5→L (14.72), H-4→L (20.34), H-3→L (48.00), H-2→L+1 (17.45), H-1→L (19.40)
	7	3.8234	H-13→L (12.64), H-6→L (39.64), H-5→L (37.72), H-4→L (20.17), H-3→L (16.28), H-2→L+1 (10.85), H-2→L+2 (10.08)
	8	3.8795	H-9→L (12.39), H-6→L (14.25), H-4→L (29.92), H-3→L (11.27), H-1→L+2 (35.95), H-1→L+4 (13.10), H-1→L+7 (10.17), H→L+2 (13.71),

Table S4. The singlet and triplet excited state transition configurations of the traditional g-C_3N_4 revealed by TD-DFT calculations. The matched excited states that contain the same orbital transition components of S_1 were highlighted in red.

	n-th	Energy (eV)	Transition configuration (%)
S_n	1	3.5043	H→L (43.51)
T_n	1	3.0843	H→L (66.08), H-1→L (14.92)
	2	3.1298	H-2→L (25.94), H-1→L (35.11), H→L+1 (25.20), H→L+1 (44.02)
	3	3.2579	H-3→L (13.92), H-2→L (18.47), H-1→L+1 (53.03), H→L+2 (29.64)
	4	3.6756	H-2→L (12.97), H-2→L+1 (23.31), H-1→L (43.38), H→L+1 (38.14), H→L+2 (16.53),
	5	3.7243	H-3→L (12.28), H-2→L (48.53), H-2→L+1 (19.25), H-1→L (17.50), H-1→L+1 (17.56), H-1→L+2 (20.77), H→L (12.76), H→L+1 (19.02)
	6	3.7996	H-2→L (13.47), H-2→L+1 (54.85), H-2→L+2 (19.68), H-1→L (24.45), H→L (10.86)

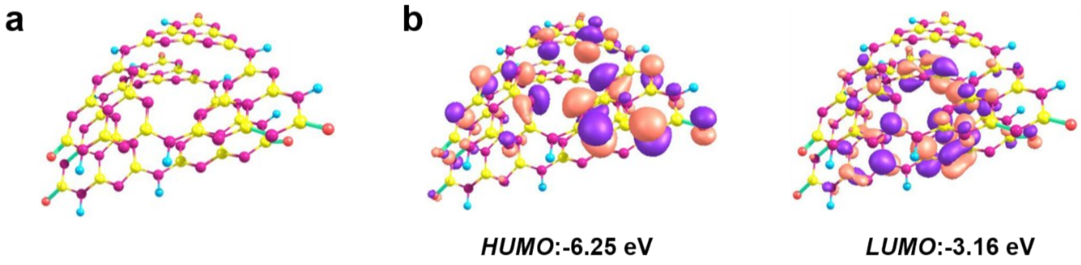

Figure S11. Optimized geometries (a) and the calculated frontier molecular orbitals (b) for the coupled W-CNQDs.

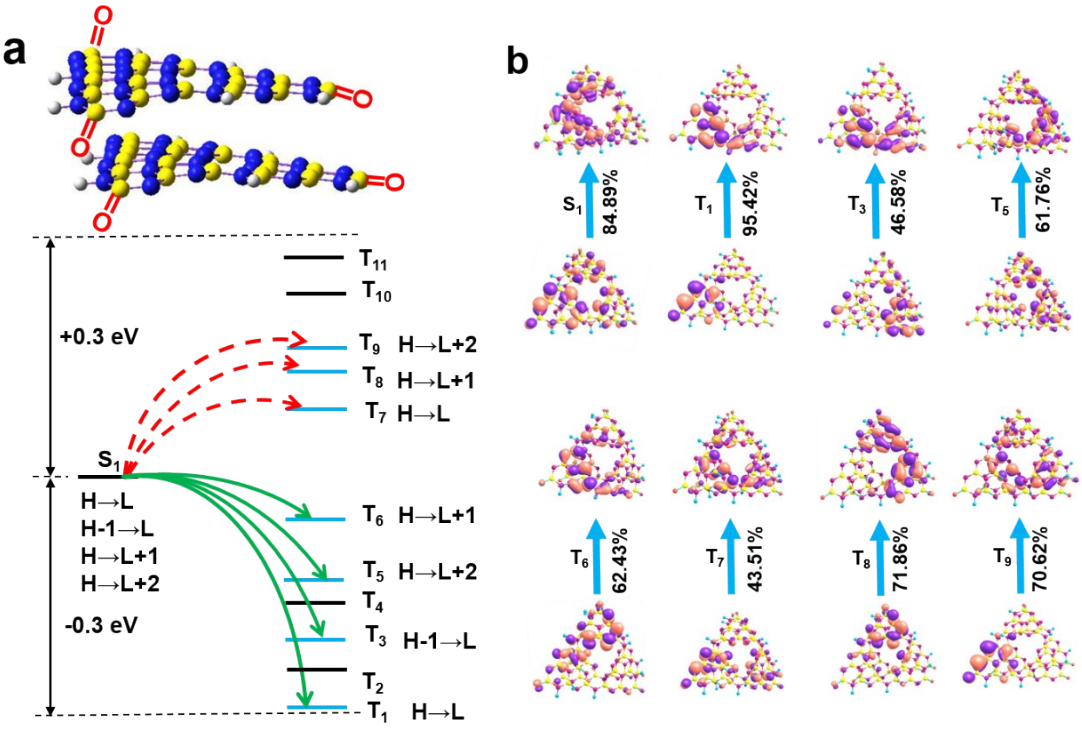

Figure S12. Theoretical calculation for phosphorescence behaviors of the coupled W-CNQDs. (a) Schematic representations of the TD-DFT calculated energy levels, main orbital configurations, and possible ISC channels at the singlet (S_1) and triplet (T_n) states. The red dashed and green solid arrows in a represent the ISC processes probably occurring from the S_1 state to its higher- or lower-lying triplet states (T_n). The blue lines refer to the triplet states available for effective ISC transitions. (b) The natural transition orbitals (NTOs) [4] (hole ones at the bottom and electron ones on the top) and the corresponding proportions.

Table S5. The singlet and triplet excited state transition configurations of the coupled W-CNQDs revealed by TD-DFT calculations. The matched excited states that contain the same orbital transition components of S_1 were highlighted in red.

	n-th	Energy (eV)	Transition configuration (%)
S_n	1	3.3946	H-1→L (13.96), H→L (48.36), H→L+1 (13.44), H→L+2 (45.56)
T_n	1	3.1054	H→L (68.78), H→L+2 (11.05), H-3→L (10.13)
	2	3.1131	H-4→L (18.68), H-3→L (65.91), H-2→L (10.51),
	3	3.1657	H-1→L (65.08), H-1→L+3 (22.08), H-3→L+4 (12.01),
	4	3.2132	H-3→L (16.62), H-2→L (15.42), H-1→L+1 (25.43), H-1→L+2 (45.23), H+2→L+1 (15.42),
	5	3.2781	H-4→L (12.91), H-3→L (18.82), H-2→L+1 (18.48), H→L+2 (49.46), H-1→L+2 (18.41),
	6	3.3241	H-5→L+1 (16.77), H-4→L (22.86), H-4→L+2 (15.08), H-3→L (10.46), H-3→L (48.00), H→L+1 (58.57)
	7	3.5216	H-5→L (35.85), H-4→L+1 (11.08), H-2→L (12.23), H→L (54.57)
	8	3.6064	H-7→L (17.58), H-6→L (27.08), H-4→L (17.94), H→L+1 (13.07), H→L+2 (52.05),
	9	3.6541	H-5→L (38.51), H-4→L+1 (37.78), H-3→L (12.13), H-3→L+1 (11.58), H-2→L+1 (21.43), H→L+2 (23.39)
	10	3.7124	H-4→L+1 (22.80), H-4→L+2 (11.51), H-2→L (28.93), H-2→L+1 (45.36), H-2→L+2 (26.72), H-2→L+3 (10.41)

	11	3.7342	H-5→L (10.33), H-3→L (26.89), H-3→L+1 (48.01), H-3→L+2 (29.66), H-3→L+4 (13.18), H-2→L+1 (12.35), H-2→L+2 (10.50)

The intermolecular electronic coupling believed to be responsible for the intensive phosphorescence emission was further confirmed by control experiments. First, when only a partial replacement of the amino groups with the carbonyl groups in the traditional g-C_3N_4 prepared from urea through the simple HNO_3/H_2SO_4 treatment as reported previously,[5] no white light but only weak blue light could be observed for the as-synthesized powder upon irradiation with a UV lamp, and no phosphorescence could be seen by naked eye under ambient conditions when the UV light was turned off. Moreover, the delayed spectra of the oxidized g-C_3N_4 showed negligible phosphorescence signals in contrast to the W-CNQDs (Figure S13). The totally different luminescence behaviors from the W-CNQDs and the oxidized g-C_3N_4 suggest that they have different intrinsic structure apart from the carbonyl groups at the rim of the π-conjugated structure. Second, when the solid powder of W-CNQDs was dispersed in water, the PL intensity decreased, but the peak position remained nearly unchanged with the main emission peak centered at 432 and 520 nm. And when the dispersed W-CNQDs in water above were reconcentrated to solid powder, the PL intensity and peak position were restored to those of the original solid powder (Figure S14), demonstrating the existence of intermolecular electronic coupling between the carbonyl groups and the large π-conjugated structure in the W-CNQDs, and for the resulting intensive phosphorescence emission. In addition, by changing the nanoparticle size for regulating the band gap of CNQDs between triplet and singlet states, blue-green fluorescence-phosphorescence dual emissive carbon nitride quantum dots (B-CNQDs) was obtained when the reaction temperature was reduced to 160 ºC without changing other reaction conditions, The carbonyl groups instead of amino groups produced at the rim of the B-CNQDs as well was verified by the optical properties and structural characterization

(Figure S15-S22, Table S6-S7), which is key to enhancing ISC and inducing intermolecular electronic coupling affording green phosphorescence.

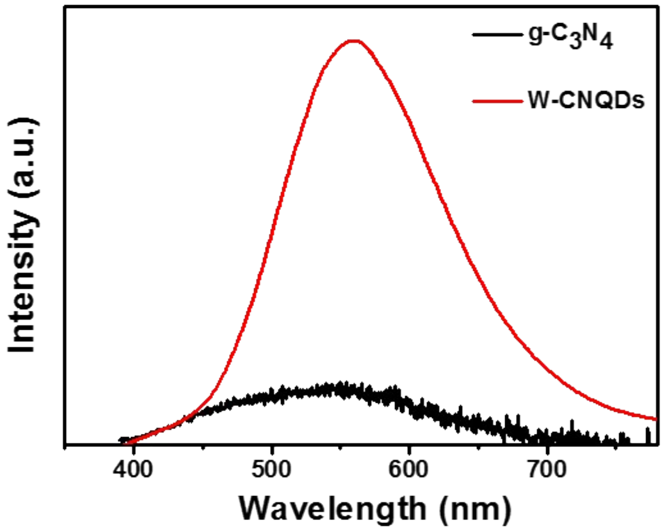

Figure S13. The delayed (5 ms) PL spectra of the oxidized g-C_3N_4 and W-CNQDs excited under 380 nm.

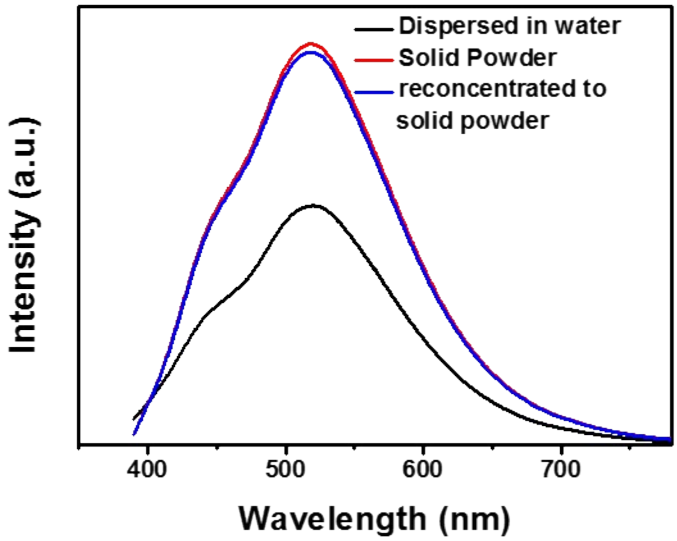

Figure S14. PL spectra of the W-CNQDs under different conditions excited under 380 nm.

The as-prepared B-CNQDs exhibit blue emission under UV (365 nm) lamp irradiation (Figure S15a) and green phosphorescence emission (Figure S15b) under ambient conditions when the UV light is turned off.

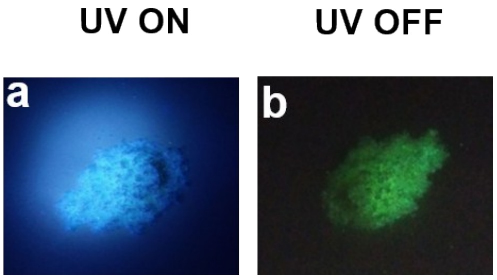

Figure S15. Photographs of the B-CNQD taken before (a) and after (b) the UV light (365 nm) is turned off.

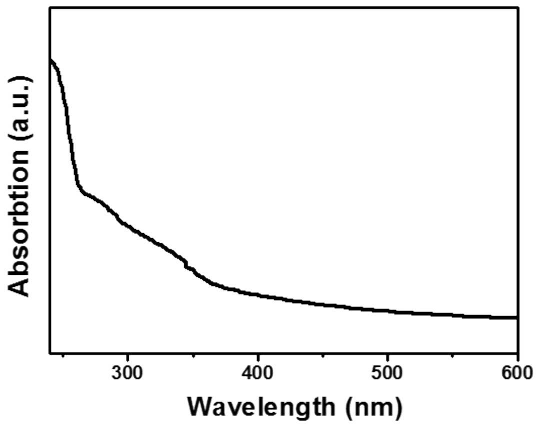

Figure S16. UV-vis absorption spectra of the B-CNQD.

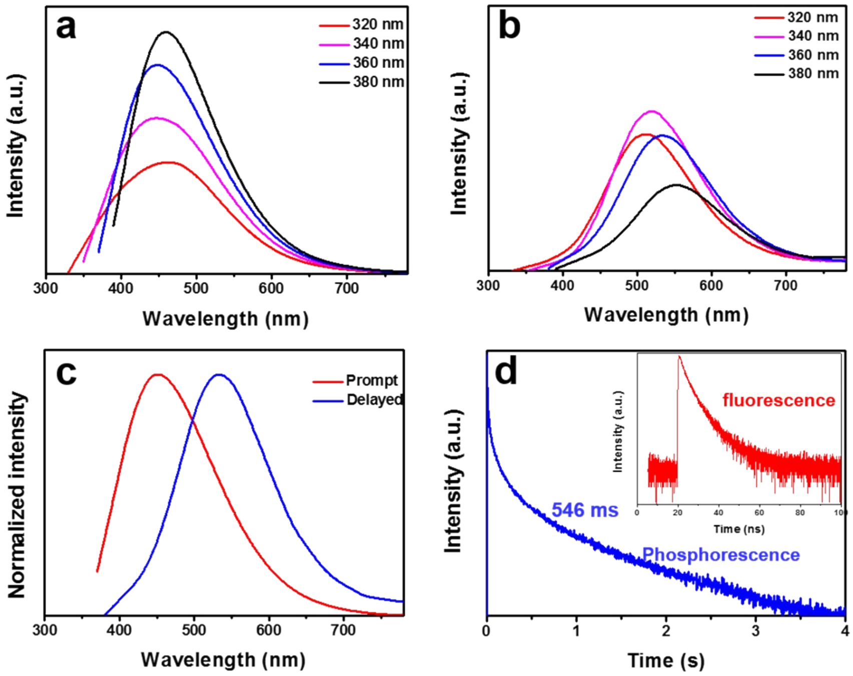

Figure S17. Photophysical properties of the B-CNQD at 295 K. (a) The prompt and (b) delayed (5 ms) PL spectra under different excitation wavelengths (c) Normalized prompt (red line) and delayed (blue line, 5 ms) PL spectra excited under 360 nm. (d) The time-resolved decay spectra measured at 456 nm for long lifetime and short lifetime (inset).

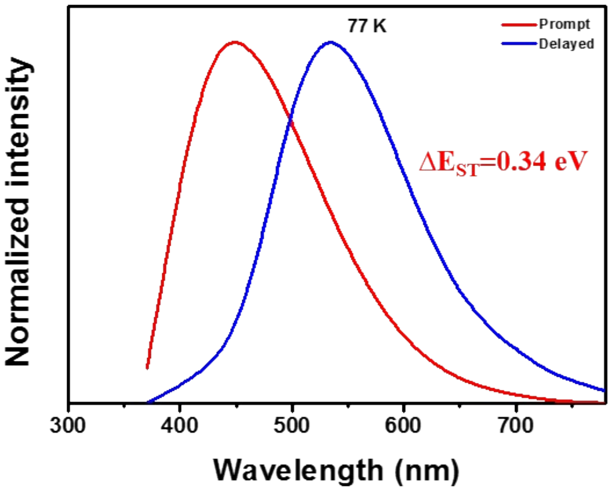

Figure S18. Normalized steady-state photoluminescence spectrum (red line) and delayed photoluminescence spectrum (blue line) of the B-CNQD excited under 360 nm at 77 K

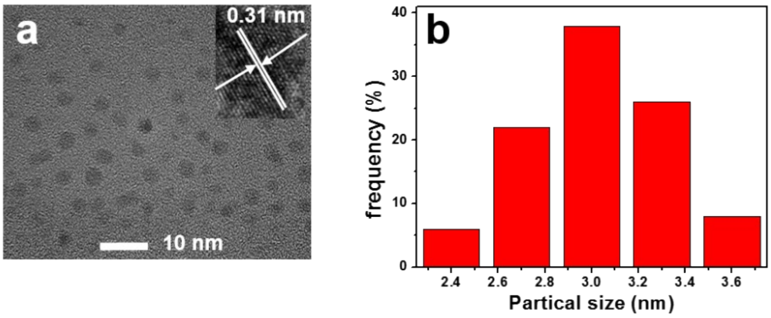

Figure S19. (a) TEM image of the B-CNQDs after ultrasonic dispersion in water (scale bar, 10 nm) (b) The size distribution of the B-CNQDs.

Table S6 Elemental analysis of the B-CNQDs.

Sample Name	C (wt %)	N (wt %)	H (wt %)	O (wt %)
W-CNQDs	33.3 %	44.4 %	3.1%	19.2%

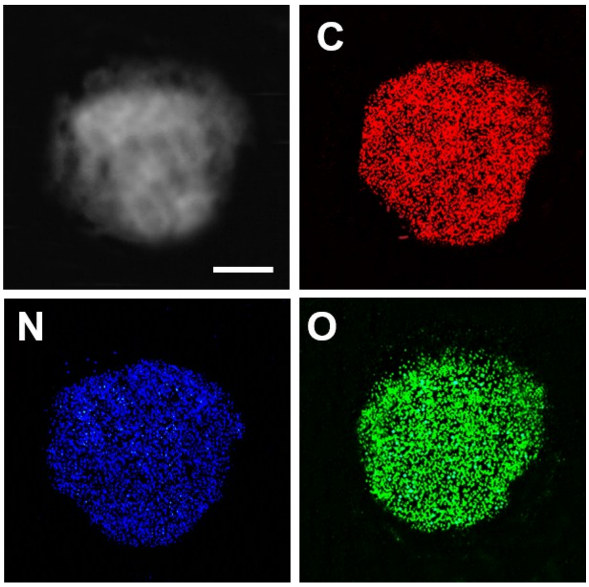

Figure S20. Elemental mapping images of the B-CNQDs (scale bar, 20 nm).

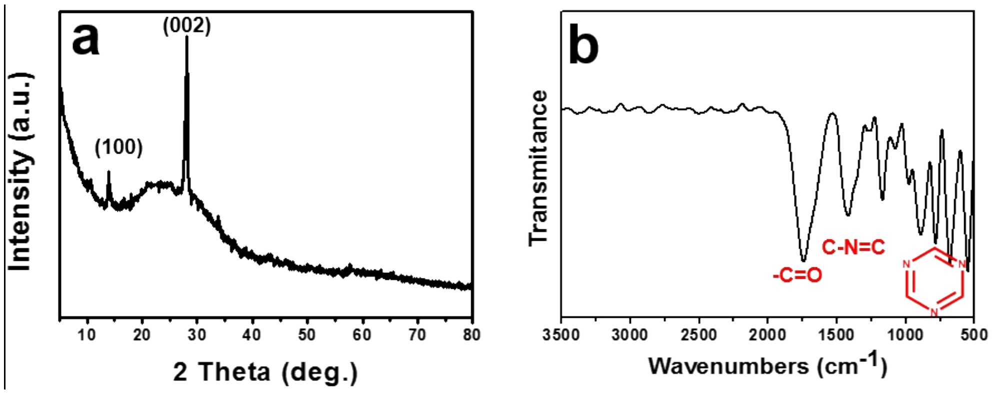

Figure S21. (a) Powder XRD patterns, (b) FT-IR spectrum of the B-CNQDs.

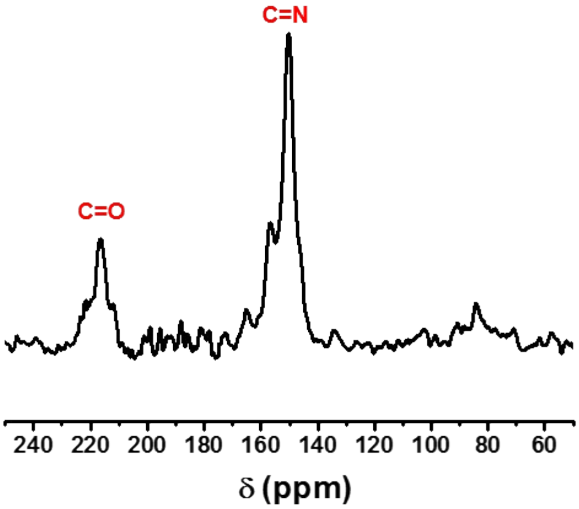

Figure S22. Solid ^{13}CNMR spectrum of the B-CNQDs.

Table S7. Dynamic photophysical parameters of the B-CNQDs.

Sample	Wavelength (nm)	Fluorescence					Phosphorescence			
		τ_F (ns)	$\emptyset_F$ (%)	k_F (s^{-1})	k_{nr}^F (s^{-1})	k_{ISC} (s^{-1})	τ_P (ms)	$\emptyset_P$ (%)	k_P (s^{-1})	k_{nr}^P (s^{-1})
**B-CNQDs**	**430**	**4.6**	**29**	**6.3** $\times 10^7$	**1.4** $\times 10^8$	**1.7** $\times 10^7$	**546**	**8**	**0.15**	**1.15**

The W-CNQDs are highly stable, and only a slight (<3%) decay was detected under continuous illumination with a λ = 365 nm UV light source for 10 h (Figure S23).

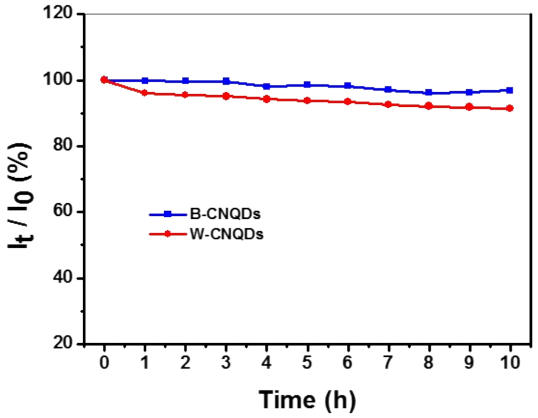

Figure S23. The photostability of the B-CNQDs, W-CNQDs under continuous radiation with a UV lamp for 10 h.

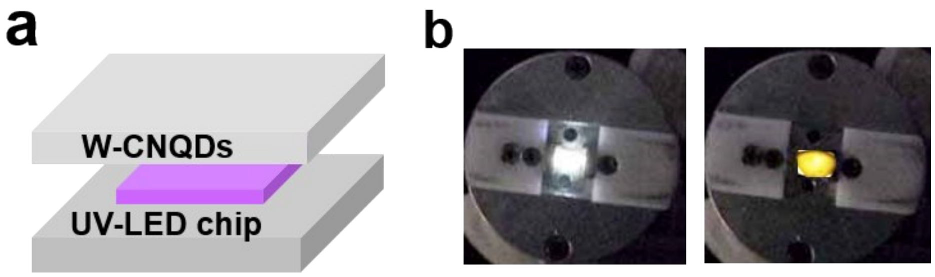

Figure S24. (a) Schematic diagram of the configuration of the prototype WLED device (b) photographs of WLED under the operation of 20 mA taken before (left) and after (right) the electrical excitation removed.

As shown in Figure S25, the EL intensity of the WLED lamp increased with enhancing the drive current from 20 to 90 mA. The CIE color coordinate, CCT and CRI showed a very slight change from (0.3510, 0.3927) to (0.3578, 0.4002), 4935 K to 4762 K, and 85 to 80.4, respectively, upon the increase of drive current from 20 to 90 mA

(Figure S26 and S27). The luminous efficiency of the WLED lamp slightly dropped from 18 to 15.2 lm/W when the drive current increased from 20 to 90 mA (Figure S28). These results show that the white light generated from the WLED lamp has high color chromatics stability against the increase of drive current, which is consistent with the high thermal stability and photostability of our CQDs phosphors. From Figure S29, the EL intensity of the WLED lamp still maintains 92% of the initial value after 72 h of continuous operation at 20 mA. The CIE color coordinate, CCT and CRI was nearly constant irrespective of the operating time (Figure S30 and S31), and the luminous efficiency only decreased about 6.1% during the first 6 h of operating and tended to remain constant from 6 to 72 h thereafter (Figure S32), demonstrating the optical stability of the single-component WLED lamp.

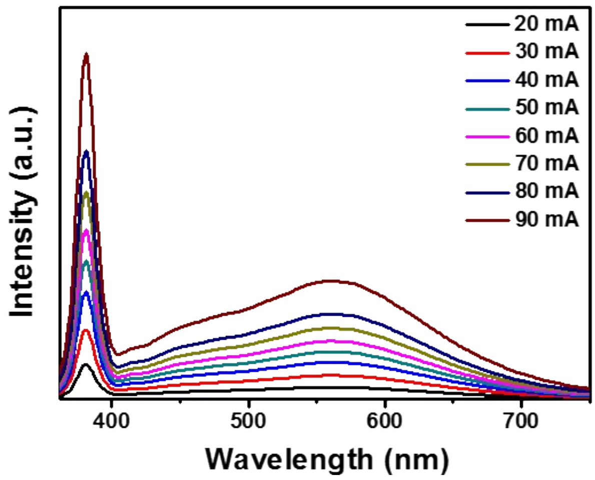

Figure S25. EL spectra of the WLED lamp under different drive currents from 20 to 90 mA.

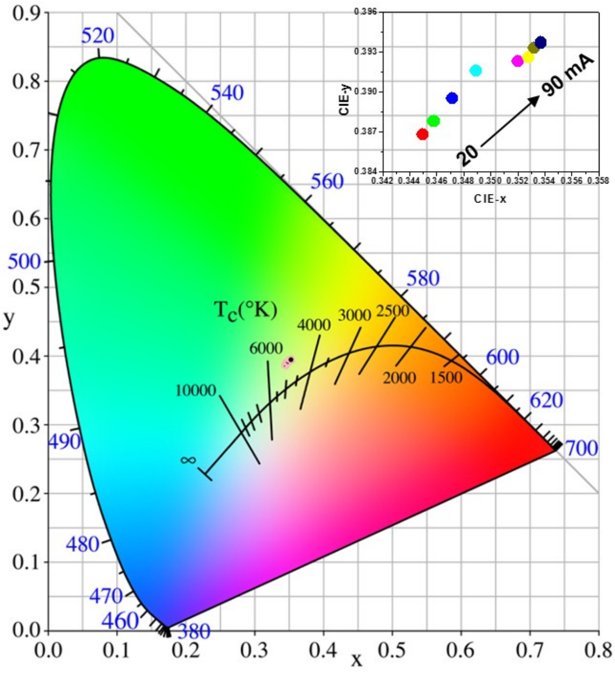

Figure S26. CIE color coordinates of the WLED lamp under different drive currents from 20 to 90 mA.

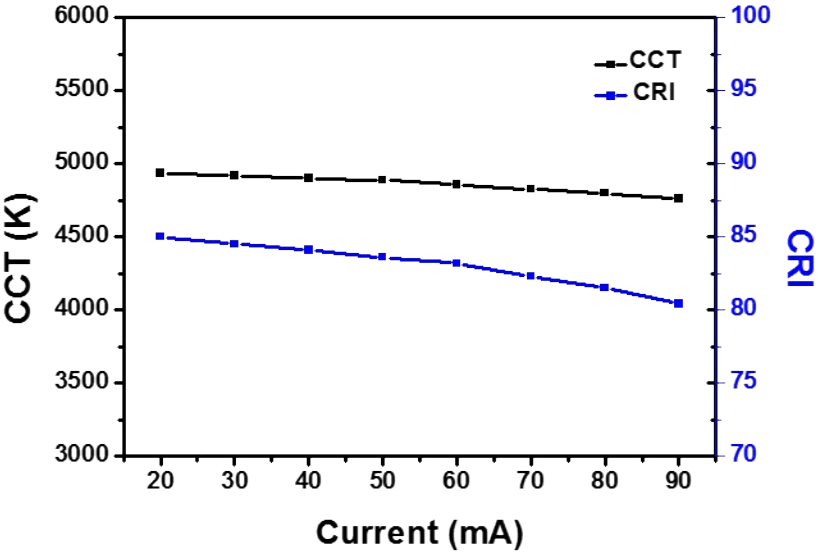

Figure S27. CCT and CRI values of the WLED lamp under different drive currents.

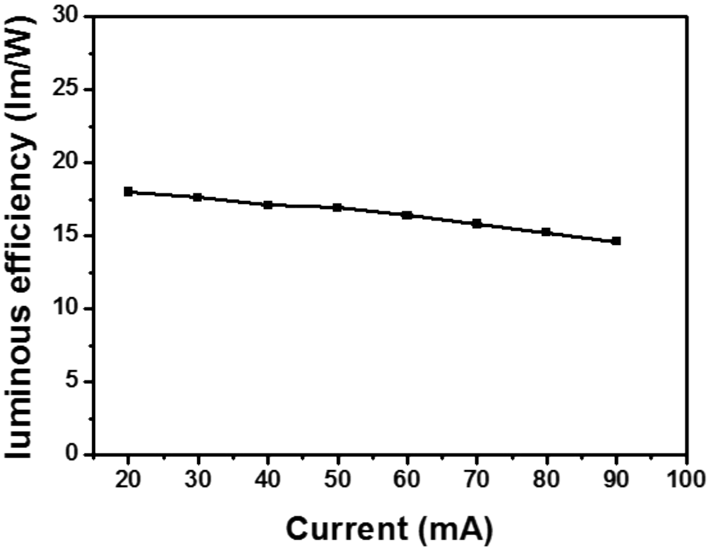

Figure S28. Luminous efficiency of the WLED lamp under different drive currents.

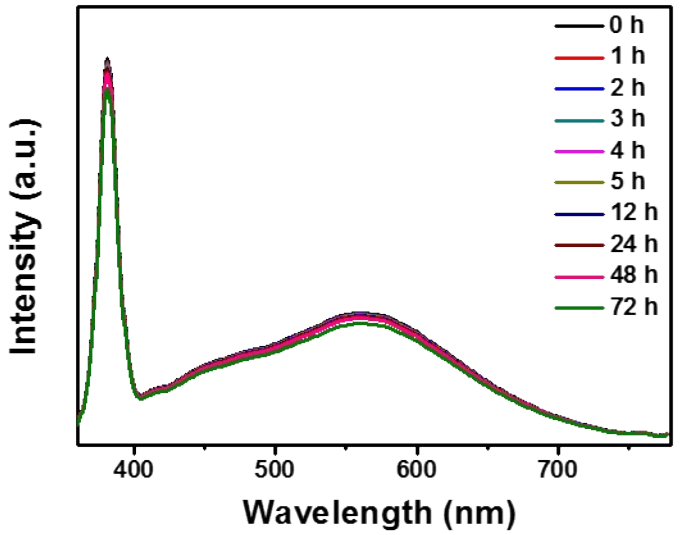

Figure S29. EL spectrum of the WLED lamp at different operating time intervals.

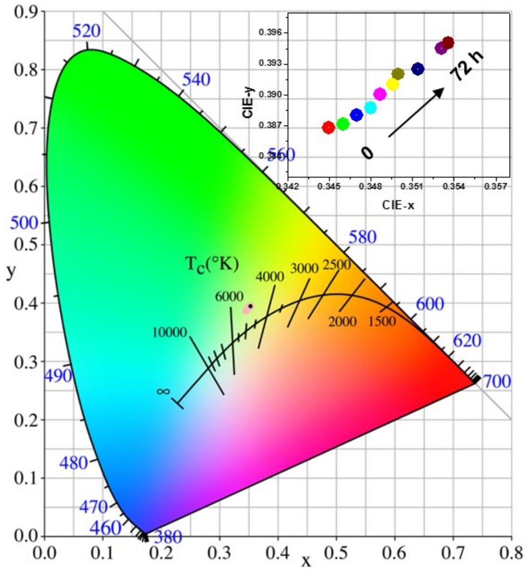

Figure S30. CIE color coordinates of the WLED lamp at different operating time intervals.

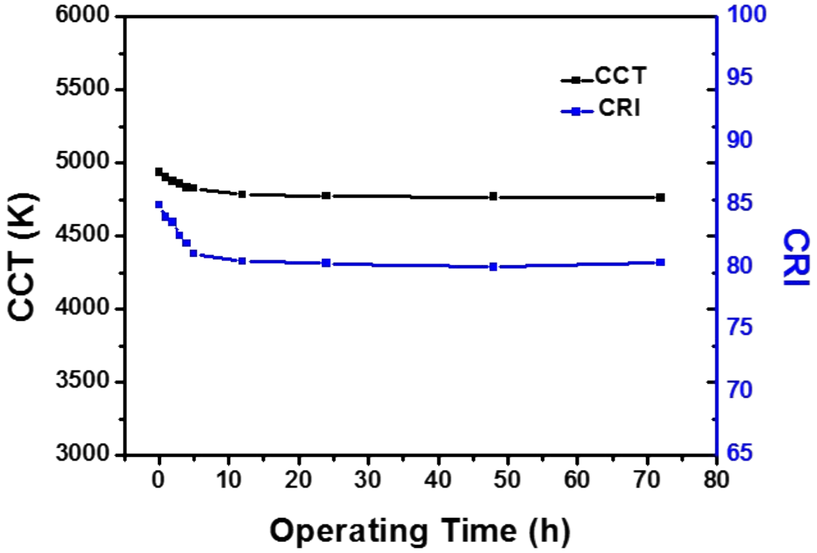

Figure S31. CCT and CRI values of the WLED lamp at different operating time intervals.

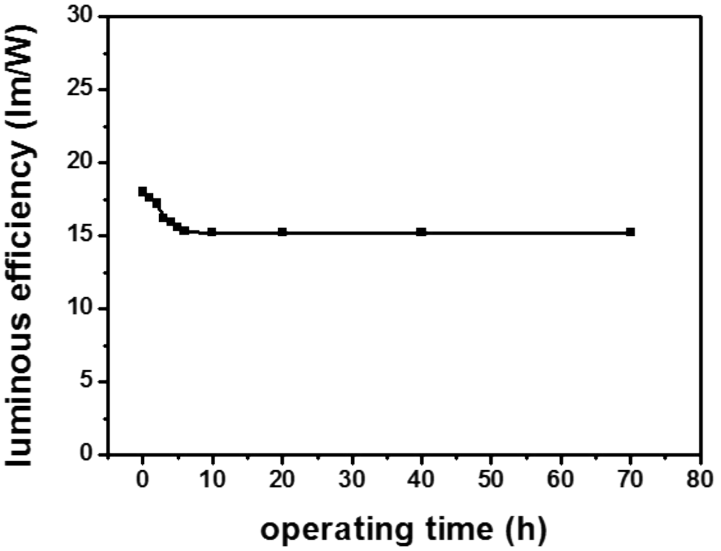

Figure S32. Luminous efficiency of the WLED lamp at different operating time intervals.

Table S8. Comparison of quantum efficiencies of WLEDs based on the W-CNQDs, lead halide perovskites and semiconductor QDs operated at 20 mA drive current.

Luminescent materials	CIE coordinates	Wavelength	CRI	QY	References
W-CNQDs	（0.35 0.39）	450 nm<br>520 nm	85.1	25%	This work
Layered perovskite	（0.39 0.42）	400 nm<br>560 nm	84	9%	[6]
two-dimensional organic−inorganic perovskite	（0.36 0.41）	420 nm<br>558 nm	82	0.5%	[7]
One-dimensional organic lead halide perovskites	（0.21 0.28）	389 nm<br>475 nm	66	20%	[8]
Ultrasmall Cadmium Selenide	（0.31 0.33）	410 nm<br>550 nm	75	8%	[9]
Magic-Sized Cadmium Selenide	（0.32 0.37）	460 nm<br>580 nm	——	2-3%	[10]
Ligand-Passivated Eu:Y_2O_3 Nanocrystals	（0.33 0.35）	520 nm	——	19%	[11]

References

[1] Y. Xiong, Z. Zhao, W. Zhao, H. Ma, Q. Peng, Z. He, X. Zhang, Y. Chen, X. He, J. Lam, B. Tang, *Angew. Chem. Int. Ed.* **2018**, *57*, 7997-8001.

[2] Z. Yang, Z. Mao, X. Zhang, D. Ou, Y. Mu, Y. Zhang, C. Zhao, S. Liu, Z. Chi, J. Xu, Y. Wu, P. Lu, A. Lien, M. Bryce, *Angew. Chem. Int. Ed.* **2016**, *128*, 2221-2225.

[3] J. A. Li, J. H. Zhou, Z. Mao, Z. L. Xie, Z. Yang, B. J. Xu, C. Liu, X. Chen, D. Y. Ren, H. Pan, G. Shi, Y. Zhang, Z. G. Chi, *Angew. Chem. Int. Ed.* **2018**, *130*, 6559-6563.

[4] T. Lu, F. Chen, *J. Comput. Chem.* **2012**, *33*, 580-592.

[5] H. Wang, S. Jiang, S. Chen, D. Li, X. Zhang, W. Shao, X. Sun, J. Xie, Z. Zhao, Q. Zhang, et al., *Adv. Mater.* **2016**, *28*, 6940-6945.

[6] E. R. Dohner, A. Jaffe, L. R. Bradshaw, H. I. Karunadasa, *J. Am. Chem. Soc.* **2014**, *136*, 13154-13157.

[7] E. R. Dohner, E. T. Hoke, H. I. Karunadasa, *J. Am. Chem. Soc.* **2014**, *136*, 1718-1721.

[8] Z. Yuan, C. K. Zhou, Y. Tian, Y. Shu, J. Messier, J. C. Wang, L. J. van de Burgt, K. Kountouriotis, Y. Xin, E. Holt, K. Schanze, R. Clark, T. Siegrist, B. W. Ma, *Nat. Commun.* **2017**, *8*, 14051.

[9] T. E. Rosson, S. M. Claiborne, J. R. McBride, B. S. Stratton, S. J. Rosenthal, *J. Am. Chem. Soc.* **2012**, *134*, 8006-8009.

[10] M. J. Bowers, J. R. McBride, S. J. Rosenthal, *J. Am. Chem. Soc.* **2005**, *127*, 15378-15379.

[11] Q. L. Dai, M. E. Foley, C. J. Breshike, A. Lita, G. F. Strouse, *J. Am. Chem. Soc.* **2011**, *133*, 15475-15486.
